# An Open Letter to the British Medical Journal

**Published:** 1878-08

**Authors:** Huntington Thomas

**Affiliations:** Chicago


### AN OPEN LETTER TO THE BRITISH MEDICAL JOURNAL.

MY DEAR BRITON :—In the number of your valuable journal, dated June 8, 1878, I find an article on the Menstrual Secretion, written by George Harley, M. D., F. R. S., to which the following note is appended :

"This article is writen acording to the new method of speling words without encumbering them with needles duplicated consonants."

On consulting further the two columns devoted to the subject named above, I find Fallopian printed *Falopian ;* call, *cal ;* attention, *atention ;* offered, *ofered ;* will, *wil ;* err, *er ;* better, *beter ;* all, *al ;* correct, *corect ;* and similar changes in every word which we have been accustomed to spell with duplicated consonants.

This admirable innovation comes to us across the Atlantic from the country which first taught us the spelling of our common language, and illustrated its form in the pages of Addison, Coleridge, Shakespeare and De Quincy. It comes, too, from the people who have habitually sneered at any orthographical change effected by Americans. I have therefore read with an interested surprise the remarkable composition of George Harley, M. D., F. R. S., and have ventured to ask you the several questions given below, upon the answers to which will largely depend an adoption of the "new method" which you have so ingeniously presented:

1. When Dr. Harley writes: "in order that the points * * *shal* be made perfectly clear," does he mean, "*may* be made perfectly clear"; or was Lord Macaulay in error when he declared that "not one Londoner in a thousand misplaces his, shall"?

2. When Dr. Harley writes: "*likely* to have their orifices ocluded," does he mean, "*liable* to have their orifices occluded;" or is the distinction between these words "an Americanism"?

3. When Dr. Harley writes: "The condition of things then found was carefuly delineated in water-colors, by a profesional artist, and stil exist in all their pristine exactitude," was he guilty of gross vulgarity and violation of the simplest rules of English grammar, or are these possibilities contemplated by the "new method"?

4. When Dr. Harley writes "presumedly" for "presumably," did he coin the word; or find it in some dictionary which has not yet crossed to these shores?

5. When Dr. Harley writes, "contrast to," for "contrast with," I desire to know if he perpetrated a Briticism?

6. When Dr. Harley writes that "the menstrual fluid consists of three *parts;*" I long to know how many "parts" have such other fluids as bile and urine.

7. When Dr. Harley writes: "although not then fuly conscious of the invaluable specimen;" was he simply not aware of its great value, or was he really rendered semi-unconscious by his "axident"?

8. When Dr. Harley writes: "and, although the woman's organs of generation had nothing whatever to do with the question of suspended animation—as I was at the time in question lecturing to the students of University Colege, on the subject of reproduction—after finishing the necropsy in as far as the pathological apearances of drowning were concerned;" I am anxious to learn whether the "new method" requires one to place his phrases as promiscuously as if they were discharged from a pepper-pot.

Many other questions are suggested by the perusal of this singular article, but I conclude with,

9. Is it well for us to make radical changes in the orthography of our mother tongue before we have learned how to write it with accuracy? In short, shall we strain at a cacography and swallow a grammar?

You are, my English cousin, the accepted journal of the British Medical Association, published in a great centre of British learning, and read probably by the larger part of the whole number of British practitioners. You have, lo, these many years, trained your batteries of criticism on medical works of American origin, and as often condemned them, even when they were of great practical value, on account of trifling blemishes in literary finish. Therefore, I cannot but regard you as of high authority in these matters. And, in the midst of my labors, which are divided between the practice of physic and the protection of my saddlebags from the attacks of the Indian and the buffalo, I write in the hope that you will enlighten my ignorance.

Fraternally yours,

HUNTINGTON THOMAS.

CHICAGO, June 27, 1878.

---

ELASTIC CRAYON OF NITRATE OF SILVER.—M. Pajot takes a laminaria tent, two millimeters in diameter, dips it into thick mucilage, and then rolls it in finely powdered nitrate of silver, and allows it to dry. He thus obtains an elastic crayon, of the ordinary size, which may be introduced into the uterus without fear of breaking. He believes this means to be applicable to other cavities, and for other more powerful caustics.

